# Resveratrol-Loaded TPGS-Resveratrol-Solid Lipid Nanoparticles for Multidrug-Resistant Therapy of Breast Cancer: *In Vivo* and *In Vitro* Study

**DOI:** 10.3389/fbioe.2021.762489

**Published:** 2021-12-07

**Authors:** Wenrui Wang, Mengyang Zhou, Yang Xu, Wei Peng, Shiwen Zhang, Rongjie Li, Han Zhang, Hui Zhang, Shumin Cheng, Youjing Wang, Xinyu Wei, Chengxu Yue, Qingling Yang, Changjie Chen

**Affiliations:** ^1^ Anhui Province Key Laboratory of Translational Cancer Research, Department of Biotechnology, Bengbu Medical College, Anhui, China; ^2^ Department of Life Sciences, Anhui Medical University, Anhui, China; ^3^ Department of Biochemistry, School of Laboratory Medicine Bengbu Medical College, Anhui, China

**Keywords:** multidrug resistance, drug delivery, resveratrol, solid lipid nanoparticle, breast cancer

## Abstract

Multidrug resistance (MDR) is a serious problem during cancer therapy. The purpose of the present study was to formulate D-α-Tocopheryl polyethylene glycol 1000 succinate-resveratrol-solid lipid nanoparticles (TPGS-Res-SLNs) to improve its therapeutic efficacy against breast cancer. In this study, the solvent injection method was used to prepare the TPGS-Res-SLNs. It was found that the TPGS-Res-SLNs exhibited zeta potential and drug-loading of −25.6 ± 1.3 mV and 32.4 ± 2.6%, respectively. Therefore, it was evident that the TPGS-Res-SLNs can increase cellular uptake of chemotherapeutic drugs, induce mitochondrial dysfunction, and augment tumor treatment efficiency by inducing apoptosis. Moreover, it was found that SKBR3/PR cells treated with TPGS-Res-SLNs exhibited significant inhibition of cell migration and invasion, as compared with free resveratrol. In addition, results from *in vivo* SKBR3/PR xenograft tumor models revealed that TPGS-Res-SLNs has better efficacy in promoting apoptosis of tumor cells owing to high therapeutic outcomes on tumors when compared with the efficacy of free resveratrol. In conclusion, the findings of the present study indicate significant potential for use of TPGS-Res-SLNs as an efficient drug delivery vehicle to overcome drug resistance in breast cancer therapy.

## Introduction

Globally, breast cancer (BC) is the second leading cause of cancer-related deaths in women (Malvezzi et al., 2019). The American Cancer Society predicts that, an estimated 281,550 new cases of invasive breast cancer will be diagnosed, resulting in 43,600 deaths in 2021 (Siegel et al., 2021). Reports indicate that genetic mutations, endocrine disorders, and a decline in immune function increases the risk of developing breast cancer (Sun et al., 2017; Sui et al., 2015; Rojas and Stuckey, 2016).

Current methods of treatment for this condition include surgery, chemotherapy, radiotherapy, or combined strategy (Untch et al., 2014; Willers et al., 1997). For instance, use of paclitaxel (PTX) has been recognized as the first-line chemotherapy in treatment of breast cancer. However, the efficacy of PTX is often limited by multidrug resistance (MDR) (Xu et al., 2016). Therefore, there is need to develop a novel strategy for overcoming this limitation and provide a safe, economically-friendly, and effective therapeutic agent against BC.

The development of MDR poses a major challenge to the existing conventional chemotherapies against BC. Functionally, an ATP-binding cassette (ABC) transporter family, comprising P-glycoprotein (P-gp), multidrug resistance-associated protein (MRP) and BC resistance protein (BCRP), utilizes ATP-derived energy to pump chemotherapy drugs out of tumor cells and protect tumor tissues from chemical toxicity (Yuan et al., 2016; Tang et al., 2016). This leads to insufficient intracellular levels of chemotherapeutic drugs and hence a poor therapeutic efficiency of the drugs. Effective reversal of P-gp-mediated MDR and maintaining accumulation of chemotherapeutic drugs in tumor tissues is imperative to management of MDR and for successful treatment of BC.

Over the past decades, considerable attempts have been made to develop P-gp inhibitors to overcome MDR. Consequently, several inhibitors, such as verapamil and disulfiram, have been identified with their co-encapsulation into nanocarriers found to reduce MDR of cancer cells *in vitro*. However, results from *in vivo* studies have been unsatisfactory, mainly because of poor aqueous solubility and low intracellular concentration (Chen et al., 2015; Alamolhodaei et al., 2017; Zhang et al., 2018). Furthermore, MDR inhibitors may have inherent toxicity on the normal cells.

D-α-Tocopheryl polyethylene glycol 1000 succinate (TPGS), as a derivative of natural vitamin E (α-tocopherol). It has been widely applied in the food and drug industry as a solubilizer, absorption enhancer, and a vehicle for nanodrug delivery system. The biological and physicochemical properties of the compound provide multiple advantages for its applications in drug delivery, including enhancement of drug solubility, improvement of drug permeation, and selective antitumor activity (Yang et al., 2018; Zhang et al., 2017; Gorain et al., 2018). Notably, TPGS can inhibit activity of ATP-dependent P-gp and act as a potent excipient for overcoming MDR in tumor tissues.

Nanotechnology has rapidly developed and plays an important role in anti-tumor, immunology as well as inflammation research (Cho, et al., 2008; Zhu et al., 2018; Zheng et al., 2019). Recently, solid lipid nanoparticles (SLNs) have attracted the most attention among all known nanocarriers in the field of drug delivery. This, owing to several unique material properties of SLNs such as small particle size, biocompatibility, chemical, and mechanical stability as well as easy functionalization potential (Banerjee et al., 2019; Shen et al., 2019; Wang et al., 2015). In addition, SLNs can modulate release of kinetics, improve blood circulation time, and increase overall therapeutic efficacy of anticancer drugs (Jiang et al., 2016). In the present study, resveratrol was successfully loaded into SLNs for treatment of breast cancer (Wang et al., 2017a). Moreover, some research studies have shown that SLNs is a promising platform for the delivery of therapeutic agents for MDR cancer chemotherapy (Majidinia et al., 2020). Based on these considerations, the current study designed TPGS-functionalized SLNs for loading drugs to overcome MDR in breast cancer.

In this study, a novel drug delivery system was developed to overcome MDR in BC, based on the findings of our previous research. First, TPGS-Res-SLNs was successfully synthesized using emulsification and low-temperature solidification methods and then characterized them using transmission electron microscopy (TEM) and zeta potential detection. The release behaviors of TPGS-Res-SLNs *in vitro* in PBS at different pH values was then measured. Second, anti-tumor efficacy of the synthesized compounds in the SKBR3/paclitaxel resistant (SKBR3/PR) cells was explored *in vitro.* The effect of these compounds on cell uptake and cytotoxicity against SKBR3/PR cells as well as anti-tumor efficacy in mice bearing an SKBR3/PR tumor were then evaluated. Further, animal studies were used to validate the treatment with TPGS-Res-SLNs, and compare the resulting effects to those of free drug. Finally, the present study examined the probable underlying mechanism of MDR inhibition in breast cancer.

## Materials and Methods

### Materials

Resveratrol, D-α-Tocopheryl polyethylene glycol 1000 succinate (TPGS) was obtained from Aladdin Chemicals (Shanghai, China). Stearic acid, lecithin chloroform, and Tween80 were acquired from Sinopharm Chemical Reagent Co, Ltd. (Shanghai, China). Dulbecco’s Modified Eagle Medium (DMEM), fetal bovine serum (FBS), penicillin G, and trypsin-EDTA were purchased from Thermo Fisher Scientific (MA, United States). Other chemicals used in this study were of analytical grade. The primary antibodies used in the experiment including P-gp, BCRP, N-cadherin, Vimentin, MMP-2, and MMP-9 were purchased from Proteintech (Beverly, MA, United States). Horseradish peroxidase (HRP) which are conjugated secondary antibodies used against rabbit or mouse immunoglobulin were purchased from Santa Cruz Biotechnology (Santa Cruz).

### Preparation of Res-SLNs and TPGS-Res-SLNs

D-α-Tocopheryl polyethylene glycol 1000 succinate-resveratrol-solid lipid nanoparticles (TPGS-Res-SLNs) were prepared using the emulsification and low-temperature solidification method. A total of 10 ml solution containing resveratrol (0.15 g), stearic acid (0.2 g), lecithin (0.1 g), and TPGS (0.1 g) were prepared in chloroform and then added into 30 ml of H_2_O containing Myrj52 (0.25 g) under fast stirring. The mixture was stirred at 1,000 rpm at 75°C for about 1 h till the total volume reduced to 5 ml, 10 ml of cold water was then added and the solution was stirred at 1,000 rpm for another 2 h at ice bath. To remove both impurities and large particles, the formulated TPGS-Res-SLNs were centrifuged at 20,000 rpm (Avanti J25 centrifuge, JA25.50 rotor, Beckman) for 1 h to collect the synthesized materials. The productive TPGS-Res-SLNs was dried for 24 h at −56°C in vacuum. Blank SLNs were developed following the same procedure without the addition of resveratrol whereas the preparation of Res-SLNs was done using the same protocol without the use of TPGS.

### Morphological, ζ-potential Characterization of Res-SLNs and TPGS-Res-SLNs

The Res-SLNs and TPGS-Res-SLNs formulation was initially imaged under TEM to investigate its morphology. A drop of a diluted suspension of Res-SLNs and TPGS-Res-SLNs formulation was placed on the carbon-coated copper grid of TEM to form a thin liquid film. This was followed by staining them with phosphotungstic acid solution (2%, w/v) for 5 min. The prepared samples were examined under a transmission electron microscope (JEOL, Tokyo, Japan). Theζ-potential of Res-SLNs and TPGS-Res-SLNs were determined using a Malvern Zeta-size Nano ZS (Malvern Instruments, Malvern, United Kingdom).

### Quantifying the Loading Efficiency of Resveratrol by UV-Vis Spectroscopy

The current study also determined the drug loading of Res-SLNs and TPGS-Res-SLNs. Briefly, 500 µl diluted Res-SLNs and TPGS-Res-SLNs formulation was placed into a 0.5 ml centrifugal Amicon^®^ filter tube (Millipore, Carrigtwohill, Ireland) and centrifuged at 14,000 rpm for 30 min at 4°C using an eppendorf centrifuge (Eppendorf AG, Germany). The loaded SLNs remained in the filter tube and the free resveratrol passed through the filter. The amount of free resveratrol in the supernatant was then quantified by UV-vis at 304 nm. The drug loading of SLNs were determined using the following equations:

$$DL\%=\frac{W_{total}-W_{free}}{W_{lipids}}\times 100\%$$

Where: *W*_free_ is the amount of resveratrol measured in the supernatant; *W*_total_ is the theoretical amount of resveratrol that was added; *W*_lipids_ is the total mass of lipids in the SLNs or in the TPGS-SLNs.

### *In Vitro* Drug Release Kinetics

To increase the solubility of resveratrol, the *in vitro* release of resveratrol from TPGS-Res-SLNs was tested using pH 7.4 and pH 5.5 buffer solutions [1:1 mixture of water and ethanol (v/v)]. Briefly, 5 ml freshly prepared TPGS-Res-SLNs solution (equivalent resveratrol dose of 30 μM) was put into dialysis tube and dialyzed against 50 ml of the medium with constant shaking at 37°C. At predetermined time points, 1 ml of the dialysis solution was removed and an equal volume of fresh medium was added. Resveratrol release was determined by measuring the absorption intensity at 304 nm using UV-visible spectrometer.

### *In Vitro* Cytotoxicity

The relative cytotoxicity of free resveratrol, Res-SLNs, and TPGS-Res-SLNs were separately evaluated *in vitro* through CCK-8 assay. The SKBR3/PR cells were seeded in 96-well plates at a density of 5 × 10^3^ cells per well in 100 μl DMEM and grew for 24 h. Subsequently, cells were incubated with free Resveratrol, Res-SLNs, and TPGS-Res-SLNs at different concentrations (0, 20, 30, and 40 μM) for 24 or 48 h. Cell viability was measured using a CCK-8 kit (Dojindo Laboratories, Japan) according to the manufacturer’s instructions and absorbance was measured at 450 nm wavelength using a spectrophotometer.

### Cell Uptake

Considering the no fluorescent property of Resveratrol, C6 was chosen as the probe for investigating the cellular uptake behavior of SKBR3/PR cells (Al-Kassas et al., 2017). The cells were seeded overnight in a 24-well plate and incubated with free C6 or C6-loaded-TPGS-SLNs at an equivalent concentration of C6 (100 ng/ml) in a medium supplement with 10% FBS. After 0.5- and 4-h incubation, the cells were washed twice with precooled PBS, fixed using 4% paraformaldehyde, and stained with DAPI dye solution for 5 min. Fluorescent images of the cells were analyzed under a TCS SP2 confocal microscope (Leica, Germany) to investigate the cellular uptake of free C6 and C6-TPGS-SLNs.

### P-gp Activity Assay

To investigate the transport activity of P-gp, Rho-123 comprising fluorescent substrates of P-gp were used (Kumar et al., 2020). Briefly, SKBR3/PR cells were pretreated with Resveratrol, Res-SLNs, and TPGS-Res-SLNs in culture medium in the dark and then co-treated with Rho-123 (0.5 μg/ml) for 1 h at 37°C. After accumulation of Rho-123, the cells were washed with ice-cold PBS and collected using trypsinization. Intracellular fluorescence of Rho-123 was measured using a FACS can flow cytometer (BD Biosciences).

### Scratch Wound Healing Assay

The wound healing assay was carried out to confirm the migration ability. The SKBR3/PR cells were first seeded in six-well plates and cultured to confluency. A single wound was created by scratching the confluent monolayer of the cell using a 10-μl sterile pipette tip. The cells were then washed two times with phosphate buffered saline (PBS) and incubated with DMEM supplemented with 1% FBS. Images were then taken using an inverted microscope at 0 and 24 h.

### Transwell Invasion Assay

The invasion assays of transfected SKBR3/PR cells were performed using Transwell cell-culture chambers (Corning, United States). The chamber was coated with the admixture of Matrigel (BD, United States). A total of 3,000 cells were the seeded into upper chamber with 100 μl serum-free medium. On the other hand, the lower chamber was supplemented with 750 μl medium containing 10% FBS. A total of 3,000 treated cells were seeded into the upper chamber with 100 μl serum-free medium and the bottom chamber was supplemented with 750 μl medium containing 10% FBS. After 24 h of incubation, these cells were washed, fixed with 4% polyoxymethylene and then stained with 0.1% crystal violet. The invaded cells were then counted and photographed under a microscope (Hanrong Company, Shanghai). Five visual fields were selected and the average number was taken.

### Cell Apoptosis

To quantitatively analyze apoptosis in SKBR3/PR cells, Annexin V-FITC/PI double staining was performed in the current study. The SKBR3/PR cells were seeded into a six-wells plate at the density of 2×10^5^ per well and cultured for 24 h. The SKBR3/PR cells were later washed with PBS (the culture medium) and was then replaced with TPGS-Res-SLNs, Res-SLNs or free resveratrol at a concentration equivalent to 30 μM Res for 24 h at 37°C and 5% CO_2_. The cells with no treatment were used as the control. Thereafter, the cells were washed with cold PBS and harvested using 0.25% trypsin (no EDTA). The collected cells were washed twice with cold PBS and resuspended in 500 μl 1×binding buffer. Five of Annexin V-Fluorescein isothiocyanate (FITC) and 5 μl Propidium Iodide (PI) were then into the resuspended cells according to the manufacture’s protocol. The plate was incubated in the dark for 30 min at 37°C before it was placed in an ice bath in preparation for flow cytometry analysis (BD Biosciences). Results were analyzed using Flowjo7.6 software.

### Cell Cycle

The SKBR3/paclitaxel resistant cells were seeded in six-wells plates at the destiny of 2 × 10^5^ per well for 24 h and then treated with TPGS-Res-SLNs, Res-SLNs or free Res for 24 h (equivalent dose of resveratrol 30 μM). The cells were then washed with cold PBS and harvested after trypsinization with 0.25% trypsin (no EDTA). Then, the cells were washed with PBS and fixed in cold 70% ethanol for 24 h. After fixation, the cells were spun again and washed with cold PBS and sequentially treated with RNase (100 μg/ml) and propidium iodide (50 μg/ml) for 20 min at 37°C in the dark. Cell cycle stages were determined and analyzed using flow cytometry.

### Immunofluorescent Staining

The SKBR3/PR cells were grown on surface-treated coverslips in 24 well plates (approximately 50,000 cells per well) for 24 h until between 50 and 60% confluence. The cells were later exposed to different formulations of Resveratrol, Res-SLNs, and TPGS-Res-SLNs (equivalent dose of free resveratrol 30 μM). After a 12-h period of drug treatment, the cells were fixed with 4% paraformaldehyde (Solarbio, Beijing, China) for 18 min, permeabilized for 8 min with 0.2% Triton X-100, and blocked for 55 min with 1% bull serum albumin (Amresco) at room temperature. The cells were probed overnight with a primary antibody against E-Cadherin (Sigma; 1:100 dilution) at 4°C and Alexa Fluor 488-conjugated goat anti rabbit IgG (Molecular Probes, Eugene, OR, 1:100) in the dark for 1 h at room temperature. After three further washes, the cells were counterstained using 4, 6-diamidine-2-phenylindole (Beyotime, 1:1000) in the dark for 5 min at room temperature. All reagents were diluted in phosphate buffered saline (PBS) with all the steps followed by three-times-5 min PBS washing. Images were captured using ZEN version 2012 software (Zeiss, Gottingen, Germany) under a laser scanning confocal microscope LSM 780 (Zeiss).

### Western Blot Analysis

The SKBR3/PR cells (approximately 5 × 10^5^) were seeded in six-well plates (cells per well) and exposed to resveratrol, Res-SLNs, or TPGS-Res-SLNs (equivalent dose of free resveratrol 30 μM) for 24 h. The medium was removed and the cells washed several times using PBS (pH 7.4) in a lysis buffer for 15 min. After centrifugation at 12,000 rpm, a BCA protein assay kit (KeyGen Biotech, Nanjing, China) was used to measure the total protein concentration of the supernatant with cell lysates. Then, equal amounts of total protein (30 μg) were analyzed on 10% SDS-polyacrylamide gel electrophoresis and transferred onto a polyvinylidene fluoride (PVDF) membranes. The samples were blocked with 5% BSA and incubated overnight with primary antibodies at 4°C. The blots were incubated with horseradish peroxidase (HRP)-coupled secondary antibody, and the bands were visualized using ImageQuant LAS4000 mini (GE Healthcare Life Science, United States). The primary antibodies used in the experiment included P-gp, BCRP, N-cadherin, Vimentin, MMP-2, MMP-9, and *β*-actin (included as a control). HRP-conjugated secondary antibodies were purchased from KeyGEN (Nanjing, China).

### Xenograft Models in Nude Mice

All animal assay were performed in accordance with the Guidelines for Care and Use of Laboratory Animals of Bengbu medical University and approved by the Animal Ethics Committee of Animal Experiments at Bengbu medical University (Permit No. SYXK Wan 2019081). Female BALB/c nude mice, aged between 5 and 6 weeks were use in this study. They were obtained by the Experimental Animal Center of the Bengbu medical college. The SKBR3/PR cells we suspended in PBS, at a concentration of 2 × 10^7^/ml. A 10 μl of the cell suspension was subcutaneously injected into the dorsal right flank of each mouse. When tumor volume reached ∼100 mm^3^, the mice were randomized into four groups as follows (*n* = 4 per group): 1) the control group: where mice were administered with a daily dose of 100 μl PBS; 2) Resveratrol group: in which mice were treated with 20 mg/kg resveratrol; 3) Resveratrol-SLNs group: where mice were treated with Resveratrol-SLNs at doses equivalent to 20 mg/kg resveratrol; 4) TPGS-Res-SLNs group: in which mice were treated with TPGS-Res-SLNs at doses equivalent to 20 mg/kg resveratrol. The tail intravenous injection is a difficult technique and it is hard to ensure the accuracy of the dose given to mice every time. Therefore, all treatments were given intraperitoneally (IP) daily for five times, to guarantee the equivalent concentration of resveratrol and improve the accuracy of this study. The shortest (S) and longest (L) diameters of the mouse tumors were measured and recorded every after 2 days using a Vernier caliper and the tumor volume (V) was calculated using the following formula:

$$\text{Tumor volume}\left(\text{V}\right)=\text{ L×}{\text{S}}^{2}/2$$

After 21 days, the mice were anesthetized, the tissues were excised, and the major organs (heart, liver, spleen, lung, and kidney) were collected for subsequent analyses.

### Immunohistochemical Staining

Expression of E-cadherin and N-cadherin was examined by immunohistochemical (IHC) staining. Briefly, paraffin-embedded tumor tissues were sliced into 3 μm sections, deparaffinized with xylene, rehydrated with alcohol gradient and then rinsed using deionized water. The tissue antigen was the retrieved by heating it for about 10 min in Tris-EDTA solution (pH 8.0), and the sections were blocked in 3% H_2_O_2_ solution. The blocked sections were incubated overnight with antibodies against E-cadherin (diluted 1:200) or Vimentin (diluted 1:100) at 4°C, washed three times with PBS, and then incubated with goat anti-rabbit IgG for 30 min. The sections were stained with freshly-prepared 3, 3′-diaminobenzidine (DAB), observed under the microscope, and pictures taken for analysis using the image processing software.

### *In Vivo* TUNEL Assay

In the current study, TUNEL assays were performed to detect DNA fragmentation, which is characteristic of apoptotic cells (Tang, et al., 2010). Tumor sections (4 μm) were deparaffinized in xylene and rehydrated in a series of graded ethanol to water. Thereafter, 50 ml of proteinase K (50 mg/ml) in TBS (pH 8.0) was added onto the slides and incubated for 30 min at room temperature. The slides were rinsed three times with PBS and then incubated for 1 h with 50 ml tunel cocktail (a mixture of 2 ml enzyme solution and 48 ml label solution) at 37°C in a humidified atmosphere in the dark. Finally, 1 μl of 1,000× DAPI stock (0.2 mg/ml) was allowed to stand in the dark for a few minutes, covered using slips and the slides analyzed under CLSM using 488 nm excitation and 530 nm emission. The cells showing green fluorescence were categorized as apoptotic. Cell nuclei staining using DAPI was also performed and the mean fluorescence intensity was measured using ImageJ software.

### Histological Examination

In the current study histopathological analysis was performed on organs and tumor tissues, using hematoxylin and eosin (H&E) staining. The main organs (heart, lung, liver, spleen, and kidney) were harvested and fixed overnight in 4% paraformaldehyde and then embedded in paraffin. The tissues were cut into 5 µm sections for H&E staining according to the standard procedures. This was followed by examination of the tissues under a light microscope (Olympus Corporation, Tokyo, Japan).

### Statistical Analysis

All results of the present study were presented as means ± standard deviation (SD). The statistical difference between two groups was tested using one-way analysis of variance (ANOVA) followed by *t*-test. The data were considered as statistically significant at *p* < 0.05 or 0.01.

## Results

### Characterization of Res-SLNs and TPGS-Res-SLNs

**FIGURE 1:**
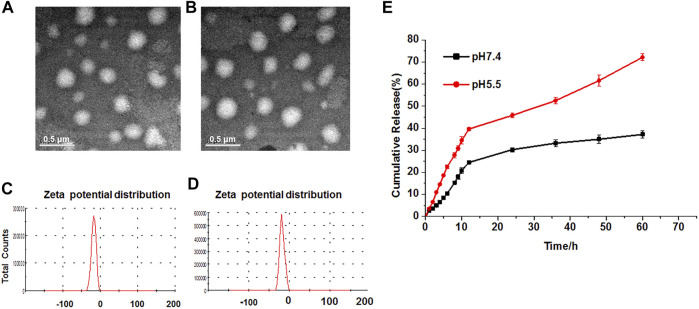
Characterization and in vitro drug release of TPGS-Res-SLNs. TEM image (scale bar: 500 nm) of **(A)** Res-SLNs and **(B)** TPGS-Res-SLNs in the suspension. The Zeta potentials of **(C)** Res-SLNs and **(D)** TPGS-Res-SLNs. **(E)** *In vitro* release profiles of Resveratrol from TPGS-Res-SLNs at different pH (pH 7.4 and pH 5.5). Data are expressed as the mean ± S.D. (*n* = 3).

Generally, the Res-SLNs and TPGS-Res-SLNs were successfully prepared in the present study. The morphology and size of these compounds were first characterized through transmission electron microscopy (TEM). Both Res- and TPGS-Res-SLNs were spherical in shape with mean diameters of 169 and 203 nm, respectively (Figures 1A,B). The zeta potential of Res-SLNs and TPGS-Res-SLNs was −27.8 ± 1.6 and −25.6 ± 1.3 mV, respectively (Figures 1C,D). The surface charge and size of nanoparticles are important parameters that affect cellular damage of cancer cells. The resultant uniform sizes of the Res-SLNs and TPGS-Res-SLNs enables better solubility and bioavailability (Bai et al., 2013).

Zeta potential is a significant factor in maintaining stability of nanoparticles in suspension through electrostatic repulsion between particles (Gao et al., 2011). In this study, zeta potential of the studied particles was negative enough to sustain the stability of TPGS-Res-SLNs dispersed system. The drug-loading of Res by TPGS-SLNs was 32.4 ± 2.6%. In addition, the release curves of resveratrol from TPGS-Res-SLNs were investigated under different pH conditions ranging from 5.5 to 7.4. Results of the current study showed that 30.27% of resveratrol was released from the TPGS-Res-SLNs at 48 h in PBS buffers (pH 7.4), whereas 45.8% of resveratrol in the PBS with pH 5.5 (Figure 1E). These results suggested that the TPGS-SLNs could be utilized for controlling the release of resveratrol in a pH-sensitive manner.

### Cytotoxicity of Res-SLNs and TPGS-Res-SLNs on SKBR3/PR

**FIGURE 2:**
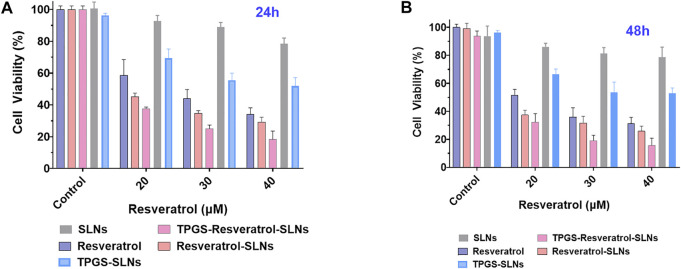
*In vitro* cell viability assay using CCK8 reagents. The cell viability of SKBR3/PR cell treated with SLNs, free resveratrol, Res-SLNs and TPGS-Res-SLNs after 24 h **(A)** and 48 h **(B)**. Data are presented as the mean ± S.D. (*n* = 3 wells). The significant difference of the results was analyzed. **P < 0.01, *P < 0.05.

To assess the potential of different drug formulations to inhibit growth of SKBR3/PR cells, a CCK8 assay was performed at 24 and 48 h. It was found that there was no toxicity caused by the nanocarrier against the SKBR3/PR cells (Figures 2A,B). After 24 h of incubation, the TPGS-Res-SLNs showed a slightly higher toxicity than free resveratrol and Res-SLNs to SKBR3/PR cells. After the incubation period, a higher cytotoxicity was recorded in TPGS-Res-SLNs relative to Res-SLNs and free Res. These results can be attributed to the fact that free resveratrol have low solubility and instability in aqueous solution. The solubility and stability of resveratrol were enhanced by loading it in SLNs or in TPGS-SLNs and this resulted in prolonged inhibition of proliferation to drug resistant cancer cells. Additionally, the rate of cell survival for TPGS-Res-SLNs was always lower than that of Res-SLNs and free resveratrol at each dose level (Figure 2).

### Efficient Cellular Uptake of TPGS-Res-SLNs

**FIGURE 3:**
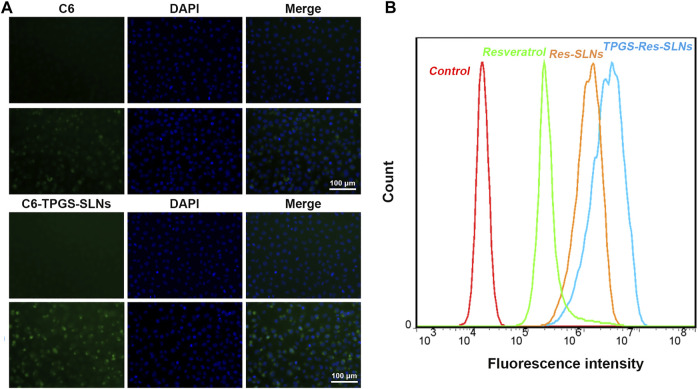
Cellular uptake and P-gp inhibition ability. **(A)** Confocal laser scanning microscopy (CLSM) images of SKBR3/PR cells after 0.5 and 4 h incubation with the coumarin-6-loaded-TPGS-SLNs (C6-TPGS-SLNs), respectively. The image was obtained from FITC channel (green), DAPI channel (blue) and the overlay of the two channels. The C6-TPGS-SLNs were green and the nucleus was stained by DAPI (blue). **(B)** P-gp inhibition ability of Resveratrol, Res-SLNs and TPGS-Res-SLNs increased uptake of Rho-123 in SKBR3/PR cells.

The uptake of different drug formulations in drug-resistant cell-line SKBR3/PR was also studied under a fluorescence microscope using the fluorescence dye, coumarin-6 (C6), and the cellular uptake was compared between C6 loaded TPGS-SLNs and coumarin-6 solution. The C6@TPGS-SLNs exhibited stronger fluorescence intensities compared to C6 after 0.5 h of incubation (Figure 3A). Thia suggested that there was a rapid internalization of C6@TPGS-SLNs into the cells within the period. Prolonging incubation time to 4 h, resulted into the entry of C6 into the SKBR/PR and into a marked increase intensity of fluorescence. When compared with C6, C6@TPGS-SLNs showed significantly stronger green fluorescence after 4 h of incubation. The results of this study indicated that uptake of C6 loaded TPGS-SLNs and C6 solution was time dependent.

### P-gp Inhibition Improves Intracellular Accumulation

The P-gp has been recognized as an active efflux transporter and plays an important role in paclitaxel resistance in various cancers including breast cancer (Che et al., 2019). To investigate the functions of free resveratrol, Res-SLNs, and TPGS-Res-SLNs on P-gp mediated drug efflux, their influence on intracellular cell uptake of Rhodamine-123 (Rho123) in SKBR3/PR cells was also assessed in the present study.

It was evident that the cells treated with free resveratrol showed significantly enhanced fluorescence relative to the control group (Figure 3B). On the other hand, the cells treated with TPGS-Res-SLNs exhibited stronger fluorescence as compared with those treated with Res-SLNs and Resveratrol. These results showed that TPGS significantly enhanced Rho123 accumulation in SKRR3/PR cells and this indicates that the inhibition of p-gp by TPGS-Res-SLNs can prevent drug efflux and increase chemosensitivity of SKBR3/PR cells.

### Effect of TPGS-Res-SLNs on Cell Cycle and Apoptosis

**FIGURE 4:**
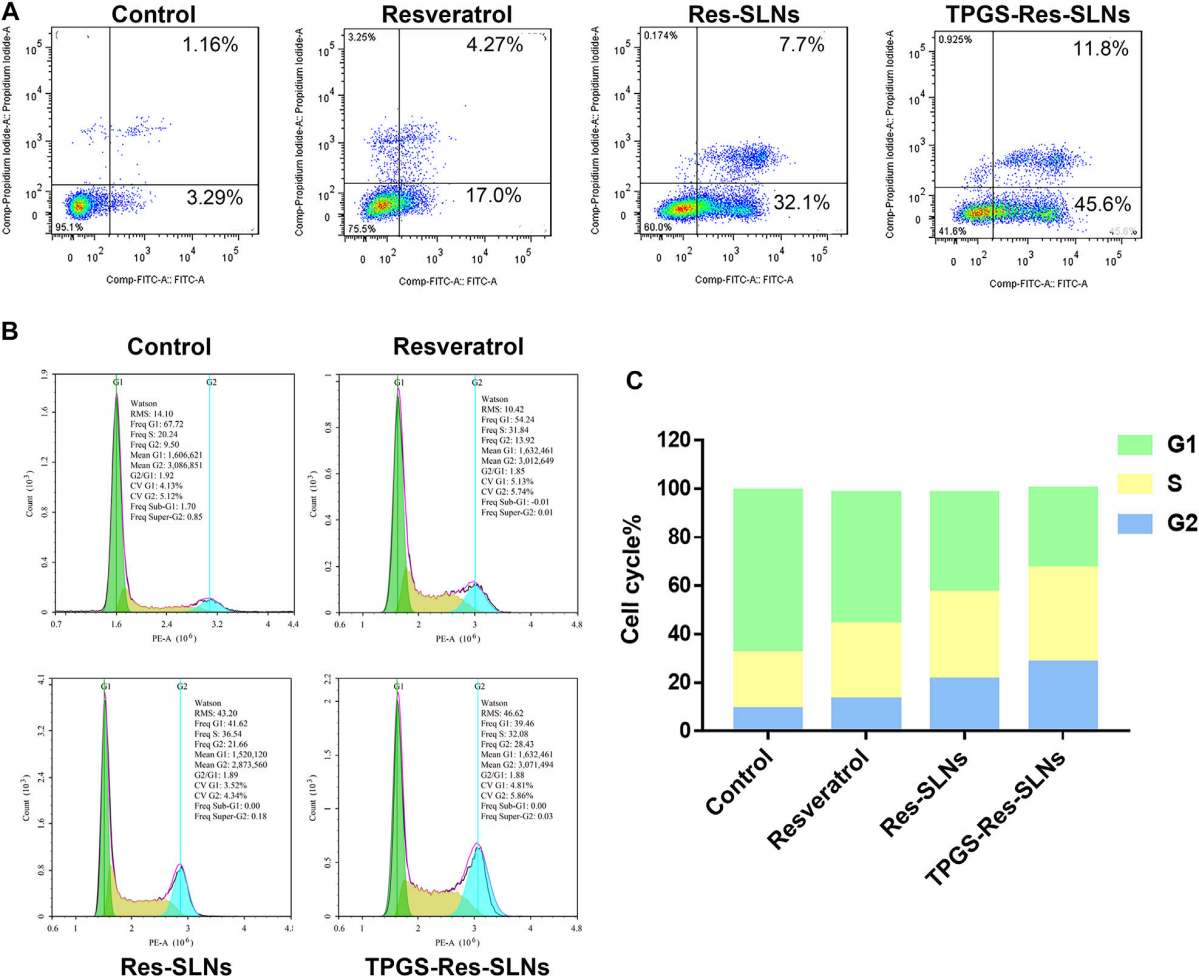
TPGS-Res-SLNs induced apoptosis of SKBR3/PR cells. **(A)** Assessment of cellular death (apoptosis and necrosis) using Annexin V-FITC/PI staining after incubation for 24 h. SKBR3/PR cells were incubated with Resveratrol, Res-SLNs and TPGS-Res-SLNs for 24 hours. **(B)** Flow cytometry analysis of cell cycle phase distribution in SKBR3/PR cells. **(C)** Histogram showing the cell cycle distribution after treatment with Resveratrol, Res-SLNs and TPGS-Res-SLNs.

To determine the anticancer effect of free resveratrol, Res-SLNs, and TPGS-Res-SLNs, the method of Annexin V-FITC & PI Double Staining was used in the present study. A 21.27% of apoptotic cells (early apoptosis 4.27% plus late apoptosis17%) was recorded in the sample incubated with free Resveratrol as compared with the untreated control group (Figure 4A). It was found that when the cells were treated with Res-SLNs, the total apoptotic SKBR3/PR cells increased to 39.80% (7.70 and 32.10% for early and late apoptosis, respectively). In addition, the percentage of total apoptotic cells treated with TPGS-Res-SLNs was 57.40%, which represented 45.60 and 11.80% for early and late apoptosis, respectively. These findings suggest that TPGS-Res-SLNs display the strongest anti-apoptotic effect in SKBR3/PR cells.

To determine the mechanism of cytotoxicity, the present study analyzed progression of the cell cycle and DNA content in SKBR3/PR cells after treatment with free resveratrol, Res-SLNs, and TPGS-Res-SLNs through flow cytometry. Incubating the cells with the three compounds revealed an increase at the G2/M phase from 9.5 to 13.92, 21.66, and 28.43%, respectively (Figures 4B,C). This indicated that all the three formulations could induce different levels of cell cycle arrest at the G2/M phase. In particular, TPGS-Res-SLNs resulted in the most significant cell cycle arrest in SKBR3/PR cells. Furthermore, these findings were consistent with the results of *in vitro* cytotoxicity.

### Effect of TPGS-Res-SLNs on Cell Migration and Invasion

**FIGURE 5:**
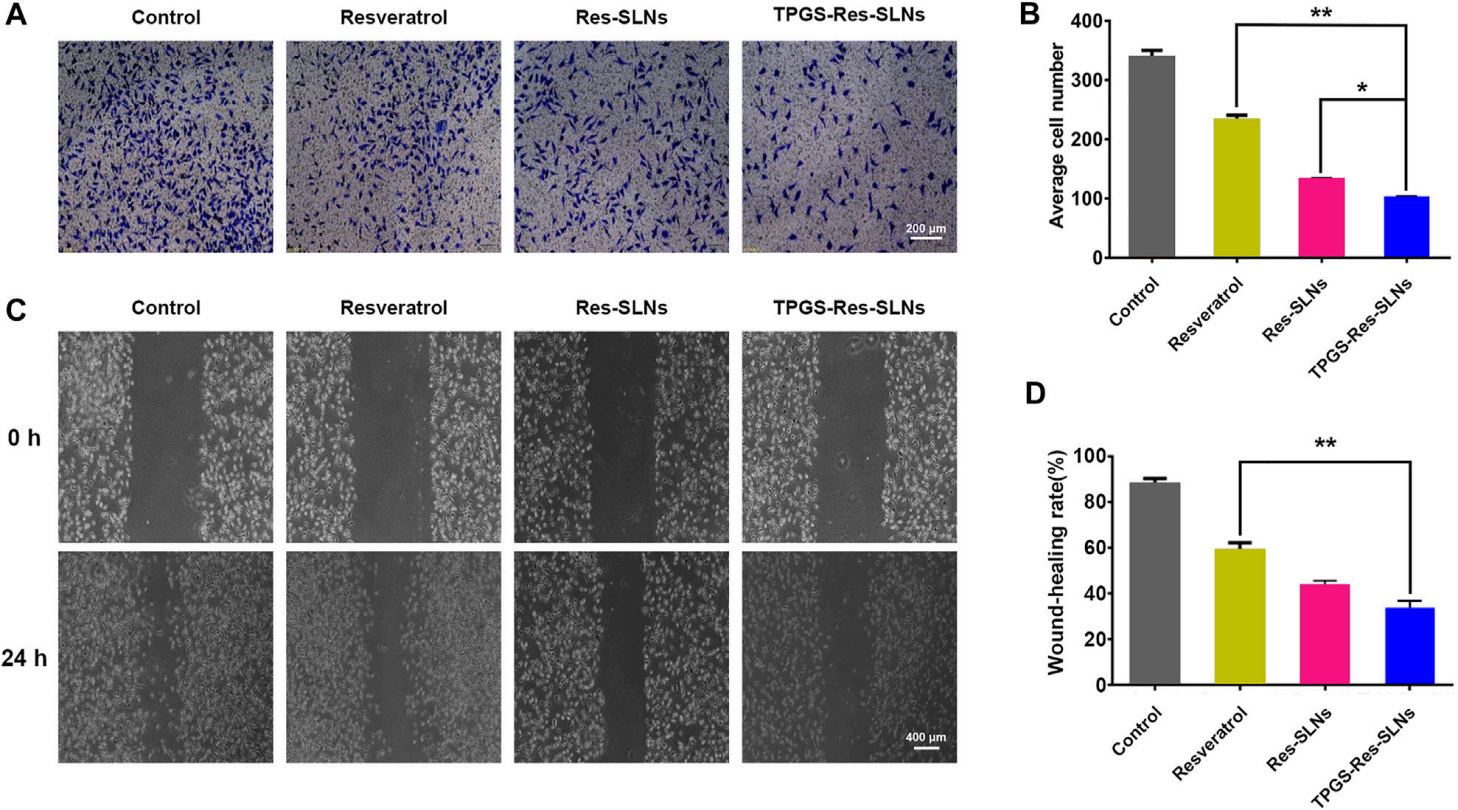
TPGS-Res-SLNs inhibited invasion and migration ability of SKBR3/PR cells *in vitro* **(A)** Transwell migration assay was used to detect the number of trans-membrane cells. Representative microphotographs of the Boyden chamber assay of SKBR3/PR cells. **(B)** The quantitative data for the Boyden chamber assay. The bar graph represents the number of invasive cells present per unit area in different treated groups. **(C)** Wound-healing assays were performed to assess cell migration. Cells were treated with Resveratrol, Res-SLNs and TPGS-Res-SLNs for 0 and 24 h or untreated. Representative images of treated and untreated cells are shown. **(D)** Distance migrated by cells at the 24 h are shown. The values represent the mean ± SD of three independent experiments. (**p < 0.01, *p < 0.05).

To further investigate whether the TPGS-Res-SLNs treatment had a negative effect on cell invasion, a transwell invasion assay was conducted using matrigel. It was found that there was a significant reduction in the number of invasive cells when treated with TPGS-Res-SLNs for 24 h as compared with when treated with Res-SLNs and the free resveratrol (Figures 5A,B).

The effect of TPGS-Res-SLNs on SKBR3/PR migration was also investigated in the current study using a wound-healing assay (Figures 5C,D). After TPGS-Res-SLNs treatment, the cell migration distance was notably shorter. This indicate that resveratrol has a negative effect on cell migration when carried by the nanoparticles. In addition, it was noted that cellular motility was also inhibited after different treatment times (0 and 24 h). The cell images and quantitative analyses revealed that there were three times fewer migratory cells in the TPGS-Res-SLNs-treated group as compared with those treated with resveratrol group.

### Mechanism by TPGS-Res-SLNs Prevents MDR

**FIGURE 6:**
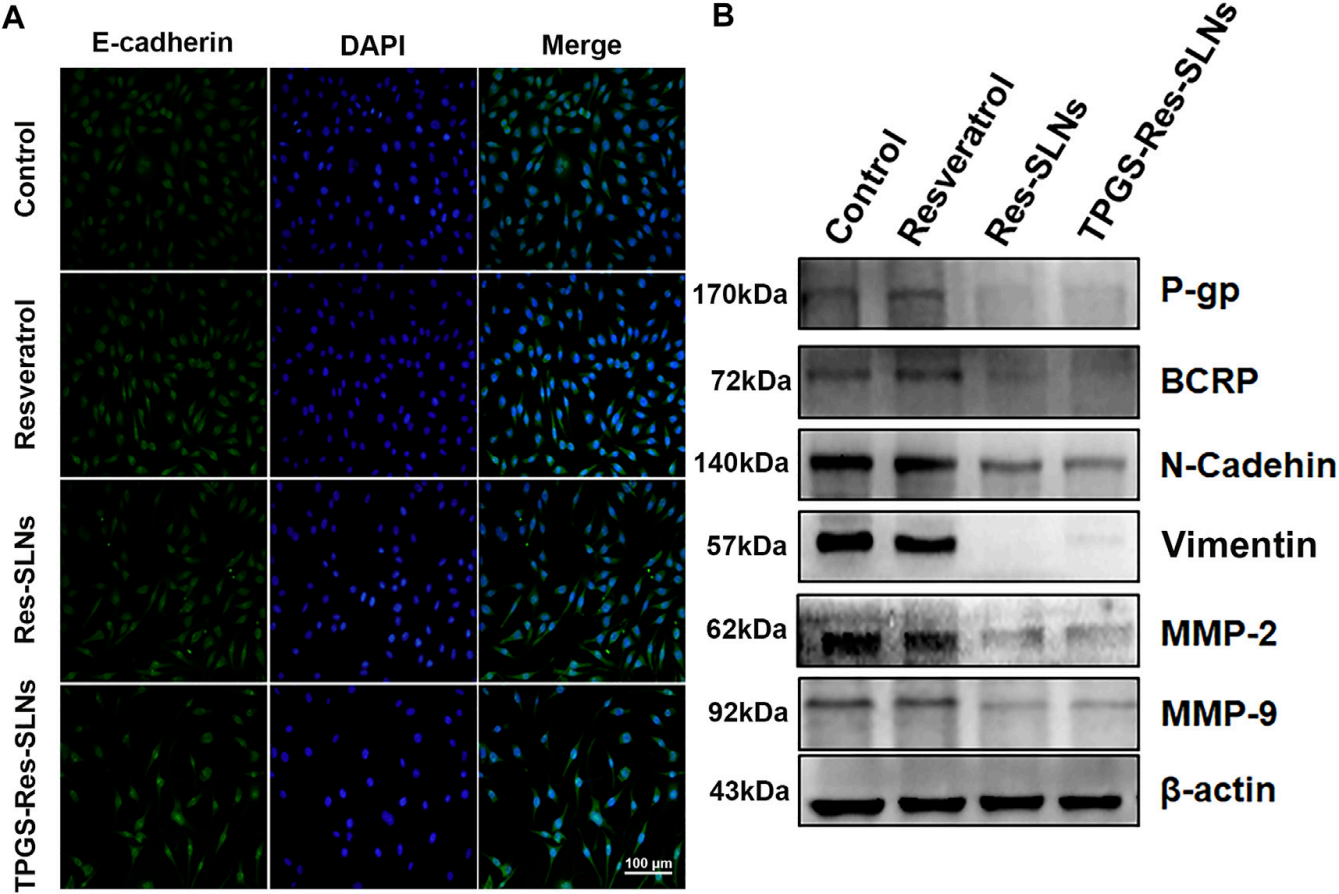
The effect of TPGS-res-SLNs on the EMT-related protein level of SKBR3/PR cells by Immunofluorescence assay and Western blot. **(A)** Immunofluorescence assay was used to detect EMT re lated proteins. SKBR3/PR cell treated with Resveratrol, Res-SLNs and TPGS-Res-SLNs for 48 h. Untreated cells served as the control. E-cadherin was stained in green, and nuclei stained with DAPI were in blue. **(B)** Expression level of P-gp, BCRP, E-cadherin, N-cadherin, Vimentin, MMP-2, and MMP-9 in protein extracted from SKBR3/PR cells analyzed by Western blot.

Previous studies have reported that SKBR3/PR cells shows enhanced migratory ability, which is associated with EMT phenotype (Smith and Bhowmick, 2016; Wang et al., 2017b; Laiolo et al., 2018). Therefore, the present study explored the effect of nano formulations on SKBR3/PR cells by transforming the EMT phenotype. Considering that EMT is characterized by a loss of epithelial markers including E-cadherin and upregulation of mesenchymal markers including N-cadherin and vimentin, the present study investigated the expressions of EMT-related proteins using western blot analysis and immunofluorescent (IF) assay. Immunofluorescence analysis revealed elevated E-cadherin protein levels were in SKBR3/PR cells (Figure 6A). Conversely, results of the western blot analysis showed that treatment with Resveratrol, Res-SLNs, and TPGS-Res-SLNs resulted in decreased levels of vimentin and N-cadherin (Figure 6B).

Multi-drug resistance (MDR) is common in patients and has been linked to overexpression of drug efflux transporters P-gp and BCRP (Chiu et al., 2016). Overexpression of P-gp is associated with resistance to a wide range of anticancer drugs, including several natural product substances such as paclitaxel (Wang et al., 2017c). Previous studies eports have implicated some nanodrugs in inhibition of MDR in tumor cells and down-regulation of P-gp expression, thus reducing drug efflux (Wang et al., 2019). Based on this, the present study used western blot analysis to investigate the effects of different nano-formulations on P-gp and BCRP expression in SKBR3/PR cells. It was evident that treatment with free resveratrol did not result in a significant decrease in P-gp and BCRP protein levels, but a significant reduction in expression of P-gp and BCRP in the two (Res-SLNs, and TPGS-Res-SLNs) drug formulations was observed (Figure 6B).

Many herbal medicines against drug resistance have been reported in multiple cancer types. Resveratrol was assessed on various types of cancers as a chemotherapy sensitizer including pancreatic cancer, breast cancer, and colon cancer. The mechanisms by which resveratrol chemosensitizes cancer cells include by inhibition of tumor cell proliferation, metastasis, and angiogenesis. Resveratrol was assessed as a chemotherapy sensitizer on various types of cancers, including pancreatic cancer, breast cancer, and colon cancer. The mechanisms by which resveratrol chemosensitizes cancer cells include by inhibition of tumor cell proliferation, metastasis, and angiogenesis.

Docetaxel–resveratrol combined treatment provides a promising future for gastric cancer patients to postpone drug resistance and prolong survival. D-α-Tocopheryl polyethylene glycol 1000 succinate can inhibit P-gp, thus sensitizing MDR cells. In the present study, the effect of SLNs with TPGS on sensitizing MDR cells was investigated in SKBR3/PR cells. Solid lipid nanoparticles serve as potential anticancer drug delivery nanocarriers, because they exhibit great superiority to modulate drug release, improve anticancer activity, and overcome MDR. Res-SLNs exhibited high cytotoxicity and allowed efficient intracellular drug delivery. This dual inhibitory strategy can have a significant potential in the clinical management of MDR in cancer.

Matrix metalloproteinases, especially MMP-2/-9, play important roles in breaking down the extracellular matrix (ECM) (Dia and Pangloli, 2017). Loss of the ECM of blood or lymph vessels allows cancer cells to invade into the blood or lymphatic system and spread to other tissues and organs. The present study evaluated whether different nano-formulations suppresses cancer cell invasion and motility by affecting the expression of matrix metalloproteinases. This was performed through a western blot analysis assay to examine the expression of MMP-2 and MMP-9 in cancer cells after treatment with different nano-formulations. Particularly, it was found that there was a significant decrease in MMP-2/MMP-9 expression in SKBR3/PR cells following treatment with resveratrol, Res-SLNs, TPGS-Res-SLNs as compared with the controls (Figure 6B). These results corroborated with the findings obtained from the transwell-based migration assay.

### *In Vivo* Anti-Tumor Efficacy in SKBR3/PR Subcutaneous Bearing Nude Mice

**FIGURE 7:**
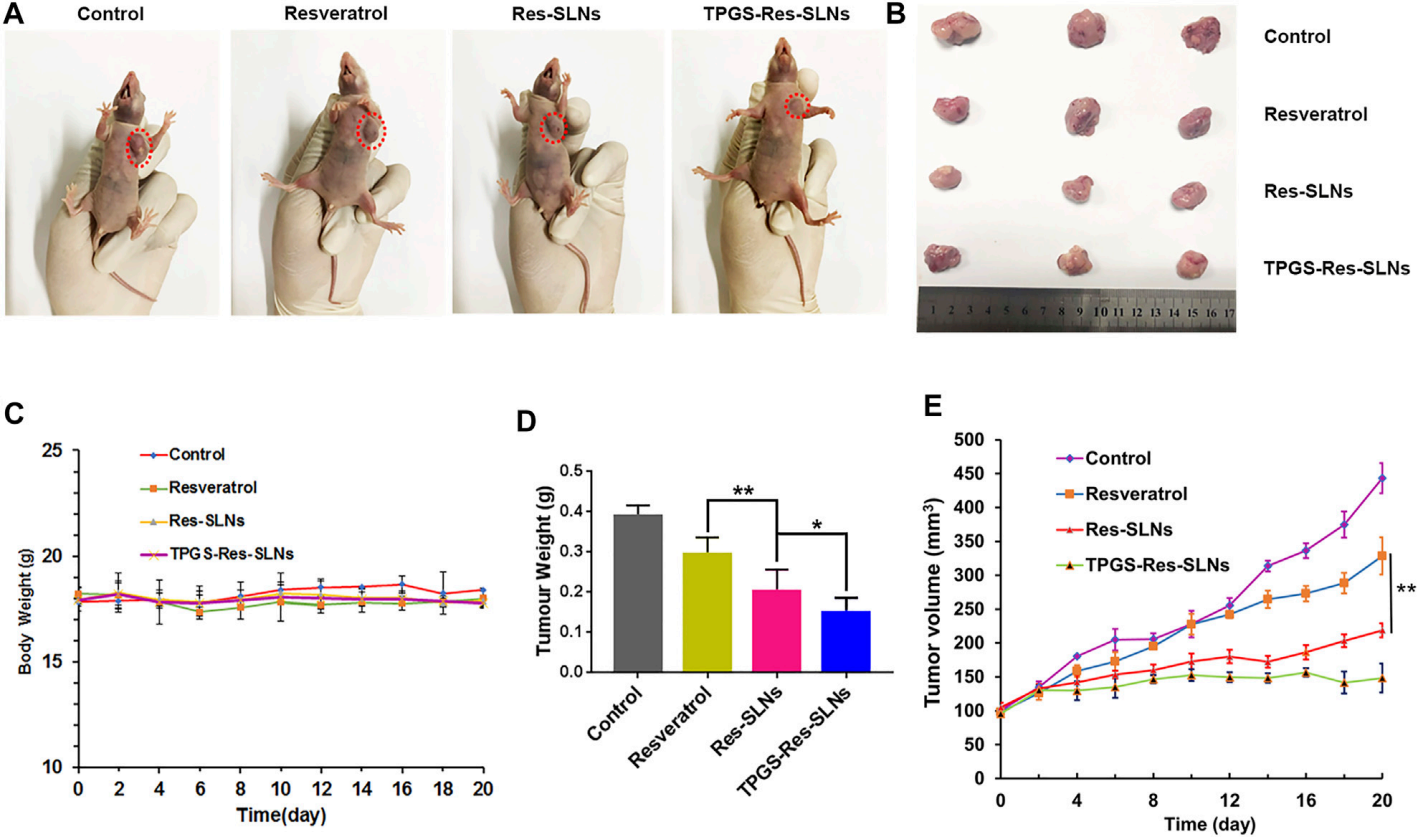
The *in vivo* efficacies of Resveratrol, Res-SLNs and TPGS-Res-SLNs on mice bearing SKBR3/PR xenografts. **(A)** Representative images of mice at the 16th day in the different treatment groups. **(B)** Digital images of tumors excised from representative mice after the indicated treatments. **(C)** Body weight vs. time curves for mice treated with the indicated formulations. **(D)** Tumor weight of mice in the different treatment groups. **(E)** Tumor volume vs. time curves for mice treated with the indicated formulations. he data represented mean ± SD (*n* = 4). *p < 0.05, **p < 0.01.

Having confirmed the good performance of TPGS-Res-SLNs through *in vitro* experiments, we further investigated the antitumor effect of the nanoparticles *in vivo* in SKBR3/PR tumor-bearing mice. Different formulations of drugs were intraperitoneally injected into the mice. Of note, the body weights of mice were not significantly altered in all treatment groups during the entire experimental period (Figure 7C). Mice treated with PBS showed faster tumor growth, whereas those treated with free resveratrol only showed slightly slower tumor growth. Comparatively, Res-SLNs and TPGS-Res-SLNs groups showed higher anti-tumor effects than groups treated with PBS and free resveratrol (Figure 7A). Images of excised tumors revealed that TPGS-Res-SLNs had a remarkably higher inhibition effect compared to other treatments (Figure 7B). In addition, mice treated with Res-SLNs and TPGS-Res-SLNs had lower tumor weight compared to control groups (Figure 7D). Analysis of tumor growth curves showed that TPGS-Res-SLNs had significantly (*p* < 0.05) higher antitumor activity than free Resveratrol and Res-SLNs (Figure 7E). These results suggested that TPGS-Res-SLNs had stronger tumor growth suppression effect due to two reasons; it had a higher cellular uptake, and prevented the inhibitory effect of TPGS on paclitaxel efflux in a P-gp-dependent manner.

**FIGURE 8:**
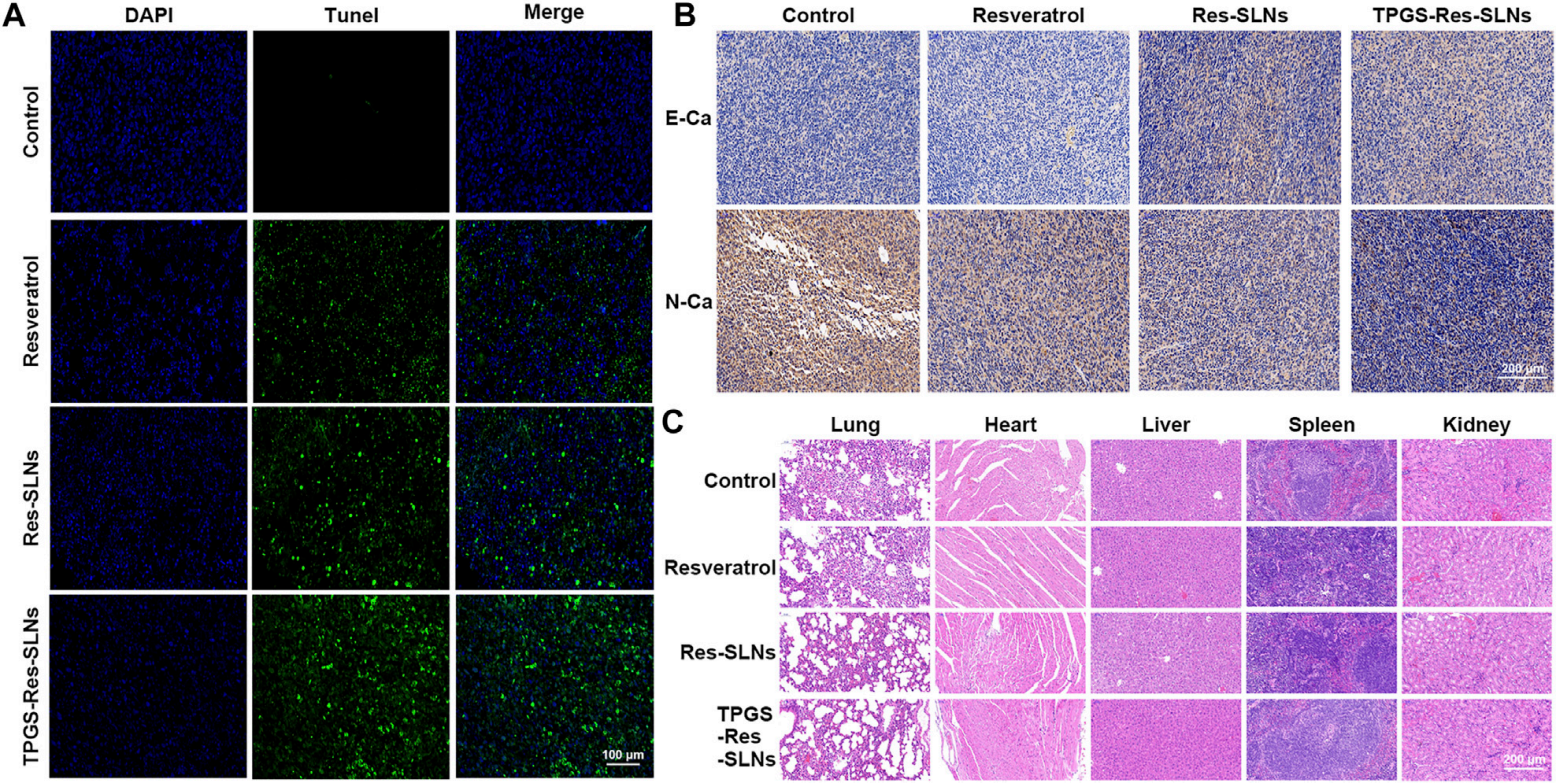
The antitumor effect of TPGS-Res-SLNs on SKBR3/PR tumor bearing BALB/c nude mice. **(A)** Apoptosis detection by TUNEL assay in slices of tumors collected from different groups of mice two days after the end of the indicated treatments. Original magnification ×400. **(B)** IHC staining of E-cadherin (E-Ca), N-cadherin (N-Ca), and positive cells of differently treated subcutaneous tumor. **(C)** H8E staining of vital organs of SKBR3-bearing nude mice after intravenous injection of PBS, Resveratrol, Res-SLNs and TPGS-Res-SLNs.

To investigate anti-tumor effects at the cellular level, the TUNEL assay was performed to analyze cell apoptosis. Similar to the tumor growth inhibition assay, we found that PBS-treated groups had no tumor cell apoptosis (Figure 8A). Groups treated with TPGS-Res-SLNs had significantly higher apoptosis compared to other groups. These findings reinforced the conclusion that TPGS-Res-SLNs had the highest therapeutic efficacy of all treatments. Taken together, these results demonstrate that TPGS-Res-SLNs can effectively load resveratrol and enhance its efficacy against breast cancer *in vivo*.

Previous studies have shown that EMT is associated with paclitaxel resistance in breast cancer cells (Tian et al., 2017). During EMT, several epithelial surface markers, primarily E-cadherin, are downregulated, whereas mesenchymal markers such as N-cadherin are upregulated. This phenomenon predominantly occurs at the invasive front (IF) of the tumor. We, therefore, examined expression of E-cadherin and N-cadherin in the tumor through immunohistochemistry. Results showed that E-cadherin expression was higher in TPGS-Res-SLNs than in free resveratrol and Res-SLNs-treated groups (Figure 8B). By contrast, N-cadherin expression was significantly lower in TPGS-Res-SLNs compared to free resveratrol and Res-SLNs treated groups.

Generally, cancer chemotherapeutics have been shown to cause severe toxicity to normal tissues. Therefore, we further explored the effect of each treatment on histopathology of major organs, heart, liver, spleen, lung, and kidney through H&E staining. It was evident that free Resveratrol, Res-SLNs, and TPGS-Res-SLNs treatments induced no obvious histological changes (Figure 8C). These results demonstrated that TPGS-Res-SLNs treatment did not induce organ damage, and can therefore be considered as a safe and effective therapeutic agent for breast cancer treatment.

## Conclusion

**FIGURE 9:**
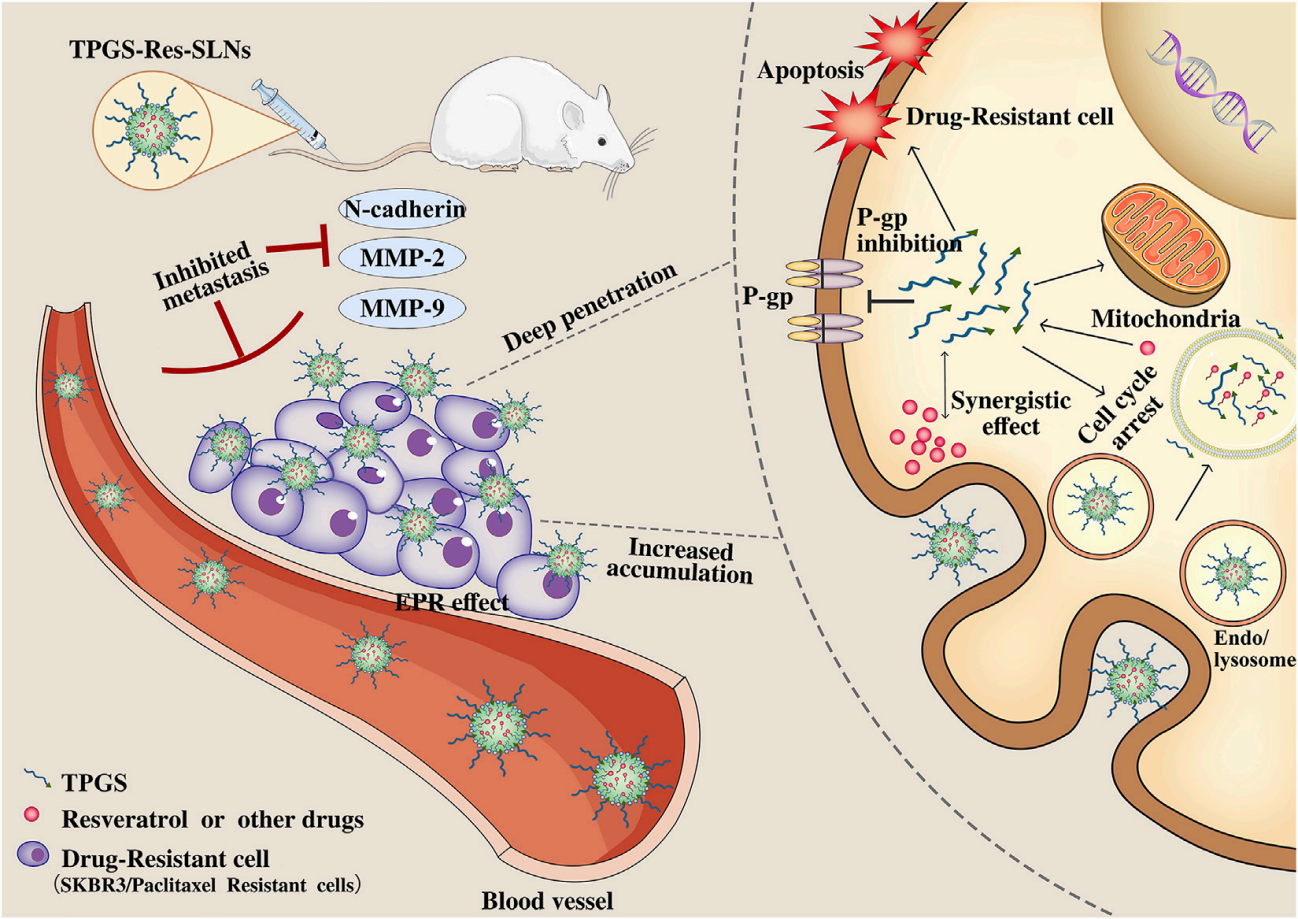
Schematic illustration of the mechanism of TPGS-Res-SLNs regulates paclitaxel resistance in breast cancer.

Although several strategies have been developed to combat multi-drug resistance in cancer cells, it remains an obstacle that limit efficacy of chemotherapy. In this study, we developed a TPGS-SLNs-based delivery strategy as illustrated in Figure 9. The TPGS-Res-SLNs and TPGS-SLNs were successfully fabricated, then loaded with Resveratrol. The morphology and size distribution of these nanoparticles were examined and their inhibitory effects on growth, apoptosis, and invasion of SKBR3/PR cells were investigated. The therapeutic effects of different formulations of these compounds were explored *in vivo* using nude mice models of SKBR3/PR, and expression of P-gp, BCRP, E-cadherin, N-cadherin, MMP-2 and MMP-9 was quantified using western blots to reveal the mechanism by which the TPGS-Res-SLNs exert their therapeutic effects. Collectively, the results demonstrated that TPGS-Res-SLNs showed great potential to treat breast cancer.

## Data Availability

The raw data supporting the conclusions of this article will be made available by the authors, without undue reservation.
